# Dose and slice thickness evaluation with nMAG gel dosimeters in computed tomography

**DOI:** 10.1038/s41598-018-21022-8

**Published:** 2018-02-08

**Authors:** Chun-Chao Chuang, Jay Wu

**Affiliations:** 1 Department of Medical Imaging and Radiological Sciences, Chung Shan Medical University, Taichung, Taiwan; 2 Department of Medical Image, Chung Shan Medical University Hospital, Taichung, Taiwan; 3 Department of Biomedical Imaging and Radiological Sciences, National Yang-Ming University, Taipei, Taiwan

## Abstract

Computed tomography (CT) has been widely used in clinical diagnosis. It is important to estimate radiation dose and perform image quality assurance procedures for CT scans. In this study, nMAG gel dosimeters were used to simultaneously measure the 300-mm weighted CT dose index (CTDI) and slice sensitivity profile (SSP) for multiple detector CT (MDCT). Magnetic resonance imaging (MRI) was performed on the irradiated gel to create R_2_‒dose response curves for the tube voltages of 120 and 140 kVp. The gel dosimeters were loaded in three home-made cylindrical phantoms to obtain CTDI_100_ and CTDI_300_. The full width at half maximum (FWHM) for 2, 5, 10, 14.4, and 38.4-mm slice thicknesses was measured and compared with the result obtained by radiochromic films. The difference in weighted CTDI_100_ obtained by the gel dosimeter and ionization chamber was less than 1%. The CTDI efficiency at 120 and 140 kVp was in the range of 80.1%–82.5%. The FWHM of SSP measured by the gel dosimeter matched very well with the nominal slice thickness. The use of nMAG gel dosimeters combined with the home-made cylindrical phantoms can provide 300-mm weighted CTDI and slice thickness information, showing potential for quality assurance and clinical applications in MDCT.

## Introduction

Computed tomography (CT) provides high-resolution cross-sectional images and has been widely used in clinical practice. According to statistical data from the National Council on Radiation Protection and Measurements (NCRP), the average effective dose for an abdomen CT scan is 10 mSv and the annual dose from CT scans accounts for 48% of total medical exposure^1,2^. Therefore, it is important to estimate radiation doses and perform image quality assurance procedures for CT scans. Even in some countries, acceptance tests and periodic quality assurance programs are required by legislation^3^.

The computed tomography dose index (CTDI) and the slice sensitivity profile (SSP) are two of the most important parameters in CT quality assurance. CTDI measurements often use a 100-mm-long cylindrical ionization chamber to integrate the radiation dose along the Z-axis during a single CT scan^4–6^. However, as the number of detector rows increases in multiple detector CT (MDCT) as well as cone beam CT (CBCT), the CTDI measurement may become insufficient to cover the entire beam width and the scatter distribution outside the collimator, resulting in an underestimation of absorbed dose^7,8^. Although a 300-mm ionization chamber could be used to increase the measurement range in the Z direction, this device is fragile and expensive, and not yet widely available for clinical use^9,10^.

In terms of SSP evaluation, the dose distribution along the Z-axis for a single CT scan is used to calculate the full width at half maximum (FWHM) as an index for longitudinal resolution. Thermoluminescent dosimeters (TLD)^11^ and photodiodes^12^ have been used for SSP measurements. Due to the limited detector size, stacking of dose points to form a two-dimensional (2D) dose profile is required. For example, Paschoal *et al*.^13^ used a high-sensitivity photodiode array with 31 aligned photodiodes to measure single scan dose profiles. The spatial resolution and the nature of 2D dosimetry make radiochromic materials a suitable choice for dose profile measurements. Li *et al*.^14^ used radiochromic films to record CT dose profiles and investigate the impact of X-ray energy and spatial resolution. Some physical phantoms for image quality assurance, e.g. the Catphan 404 slice geometry module, use a shallow-angle slice ramp to measure the cross-plane spatial resolution^15^. However, these phantoms cannot be used for measuring the dose distribution and beam width for MDCT or CBCT.

In recent years, gel dosimeters are gradually being employed for dose assessment^16–18^ in medical applications. Gel dosimeters provide three-dimensional (3D) dose measurements and have a number of exceptional characteristics such as tissue equivalence, high spatial resolution, low energy dependence, and low dose rate dependence. In this study, a methacrylic acid-based gel (nMAG) dosimeter was used to simultaneously assess radiation dose and longitudinal resolution of CT scans. For dose evaluation, the integration length of CTDI was extended to 300 mm by using home-made PMMA phantoms. For SSP evaluation, the dose distribution along the Z-axis was measured using the nMAG gel and FWHM for different slice thicknesses was calculated.

## Materials and Methods

### CTDI algorithm

The International Electrotechnical Commission (IEC) defines the CTDI_100_ as an integral of dose distribution over 100 mm along the Z-axis divided by the product of slice thickness (*T*) and slice number (*n*):

1 $${{\rm{CTDI}}}_{100}=\frac{1}{nT}{\int }_{-50\,mm}^{+50\,mm}D(z)dz.$$

A pencil-type ionization chamber (WDCT 10, RTI Electronics AB, Sweden) with the effective length of 10 cm and the diameter of 0.9 cm was used to measure the CTDI_100_ in the center and the periphery of a commercial CTDI phantom. The weighted CTDI is calculated as follows:

2 $${{\rm{CTDI}}}_{{\rm{w}}}=\frac{1}{3}{{\rm{CTDI}}}_{{\rm{c}}}+\frac{2}{3}{{\rm{CTDI}}}_{{\rm{p}}},$$

where CTDI_c_ and CTDI_p_ are the CTDI measured in the central and peripheral regions of the CTDI phantom, respectively.

In this study, home-made PMMA cylindrical phantoms and nMAG gel dosimeters were used to extend the measurement range along the Z-axis to 450 mm. The radiation dose within 300 mm was integrated to obtain CTDI_300_. The ratio of weighted CTDI for 100-mm integration length to that for 300-mm defines the CTDI efficiency as

3 $${{\rm{CTDI}}}_{{\rm{eff}}}={{\rm{CTDI}}}_{100,{\rm{w}}}/{{\rm{CTDI}}}_{300,{\rm{w}}}.$$

### Preparation of gel dosimeters

The nMAG gel dosimeters were prepared according to the recipe proposed by Karlsson *et al*.^19^ in a normal oxygen environment. 8% gelatin (300 Bloom Type A, Sigma-Aldrich, St Louis, MO) was mixed with 84% pure water and heated to 45 °C until the gelatin was completely dissolved. The solution was then cooled to 32 °C, and 8% methacrylic acid (MAA, 99%, Sigma-Aldrich, St Louis, MO) was added and stirred for 25 min. Tetrakis(hydroxymethyl)phosphonium chloride (THPC) (TCI, Sigma-Aldrich, St Louis, MO) was added as a deoxidant. The solution was evenly mixed before dispensing to PMMA tubes of 16-mm diameter and 15-mm length. The gel tubes were coated with tin foil to avoid polymerization induced by external light and placed in a refrigerator at 4 °C for solidification.

### Obtaining dose response curves for CT scans

The CT scanning parameters were as follows: tube voltages of 120 and 140 kVp, tube current of 320 mAs, and slice thickness of 10 mm. Gel tubes were placed on the scanning couch, and 10–25 repeated scans were performed in the axial mode. The point dose was also measured using a Famer-type ionization chamber (FC65-P, Scanditronix Wellhofer North America, USA) under the same scanning conditions. After irradiation, the gel tubes were immediately placed in the magnetic resonance imaging (MRI) room for 24 hours of temperature equilibrium.

### MRI readouts

A 1.5 T MRI system (Siemens Sonata, Siemens Medical Solutions Erlangen, Germany) with an 8-channel head receive coil was used to read the nMAG gel dosimeters. The gel tubes were loaded in a cylindrical water phantom^20^. The multiple spin echo pulse sequence was applied to achieve 32 spin echoes. The scanning parameters were as follows: repetition time of 4500 ms, echo spacing of 22 ms, pixel size of 1 × 1 mm^2^, and field of view of 250 × 250 mm^2^. The slice thickness for the 7.7-cm center region of the gel tube was 0.7 mm, while the thickness for the outer region of the tube was 2.5 mm. After acquiring 32 sets of T_2_ images, the spin-spin relaxation rate R_2_ was estimated pixel by pixel by applying a monoexponential decay model and parametric fitting on the series of echo signals. A circular region of interest (ROI) with the diameter of 6 mm was selected at the center of the gel tube, and the mean R_2_ value was calculated to produce the dose response curve.

### CTDI measurements

**Figure 1:**
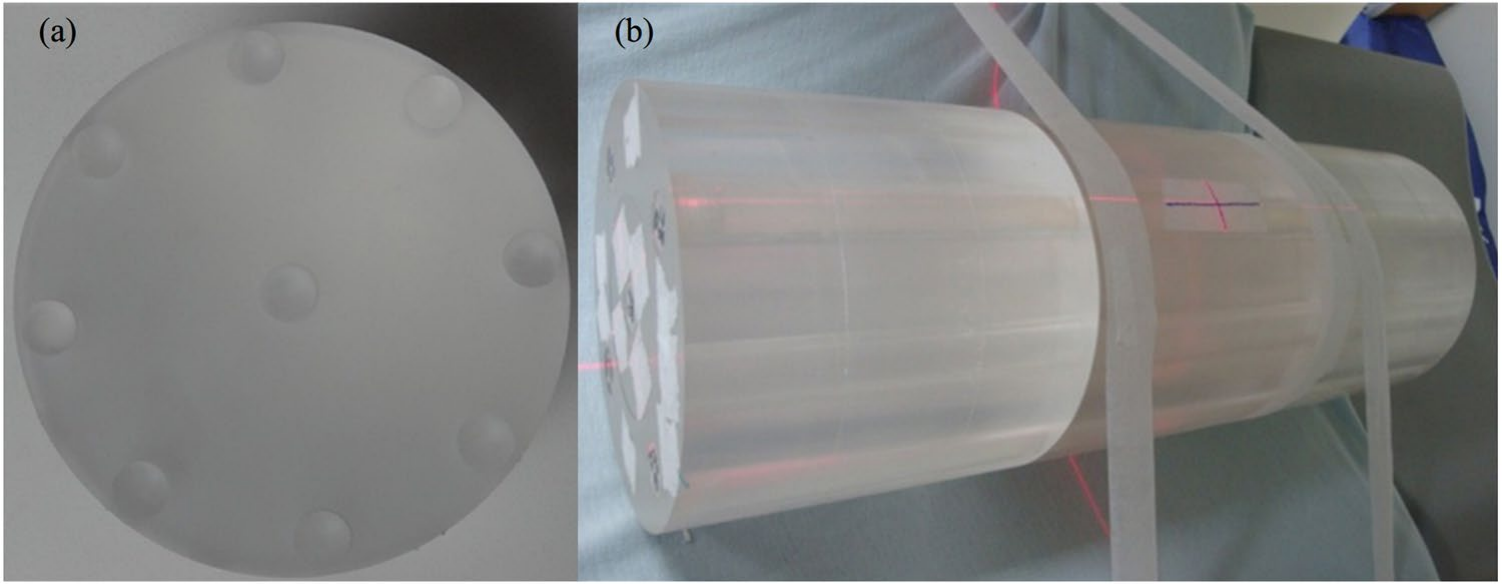
Home-made PMMA cylindrical phantoms. (**a**) The phantom has the diameter of 160 mm, length of 150 mm, and hole diameter of 16 mm. (**b**) Three phantoms were placed side by side to measure CTDI_300_ using nMAG gel tubes.

The nMAG gel tubes were loaded into a home-made PMMA cylindrical phantom with the diameter of 160 mm, length of 150 mm, and hole diameter of 16 mm. The locations of the holes were consistent with those in the 16-cm commercial CTDI phantom. Three cylindrical phantoms were placed contiguously on the examination couch to achieve the dose measurement of 450 mm (Fig. 1).

CT scans (SOMATOM Definition Flash, Siemens Healthcare, Forchheim, Germany) were performed with the following parameters: tube voltages of 120 and 140 kVp, tube current of 320 mAs, slice thicknesses of 2, 5, 10, 14.4, and 38.4 mm, and number of repeated scans of 25. After 24 hours of polymerization, the gel tubes were read by MRI. The R_2_ values along the axis of the gel tube were calculated and converted to the dose per scan using the dose response curve described above. Furthermore, the dose profile was integrated to obtain CTDI_100_ and CTDI_300_ in the central and peripheral regions of the phantoms. In addition, the 100-mm ionization chamber wrapped up by a 5-mm-thick tissue-equivalent bolus was inserted in the holes of the home-made cylindrical phantom to measure CTDI_100_ under the same scanning parameters.

### SSP measurements

Three cylindrical phantoms loaded with gel tubes were placed contiguously for CT imaging under 120 kVp, 320 mAs, and different slice thicknesses. 25 repeated scans were performed to reduce noise in the Z-axis. After the MRI readouts, the R_2_ distribution along the Z-axis was measured. Background subtraction was performed using an unirradiated gel. It is worth noting that the area within 1 cm of the tube sealing is an inactive region due to residual air. An exponential function was used to interpolate the data in this region. The 400-mm R_2_ profile was used to calculate FWHM. In addition, Gafchromic EBT film strips (International Specialty Products, NJ, USA) were placed in the phantoms to measure the dose profile under the same conditions. The strips were scanned using an Epson Expression 10000XL flatbed scanner, and the grayscale intensity along the Z-axis was directly analyzed for FWHM calculation. The FWHM results of nMAG gels and Gafchromic films were compared.

### Uncertainty

The uncertainty of dose and slice thickness measurement assessed with dosimeters can be separated into two types, A and B. The type A uncertainty lies in the statistical distribution of the repeated measurement results. The sources of type A uncertainty in nMAG gel dosimeters include the following: preparation and preservation of the gels, time delay of MRI readings, uniformity of magnetic field, errors in R_2_ fitting, CT output variations, etc. As for the type B uncertainty, it is related to the available information rather than to statistical methods. In our experiment, the total uncertainty of the ionization chamber was 0.5%, and the total uncertainty of Gafchromic films and nMAG gel dosimeters was 5%.

### Data availability

The datasets generated during and/or analysed during the current study are available from the corresponding author.

## Results

### Dose response curves

**Figure 2:**
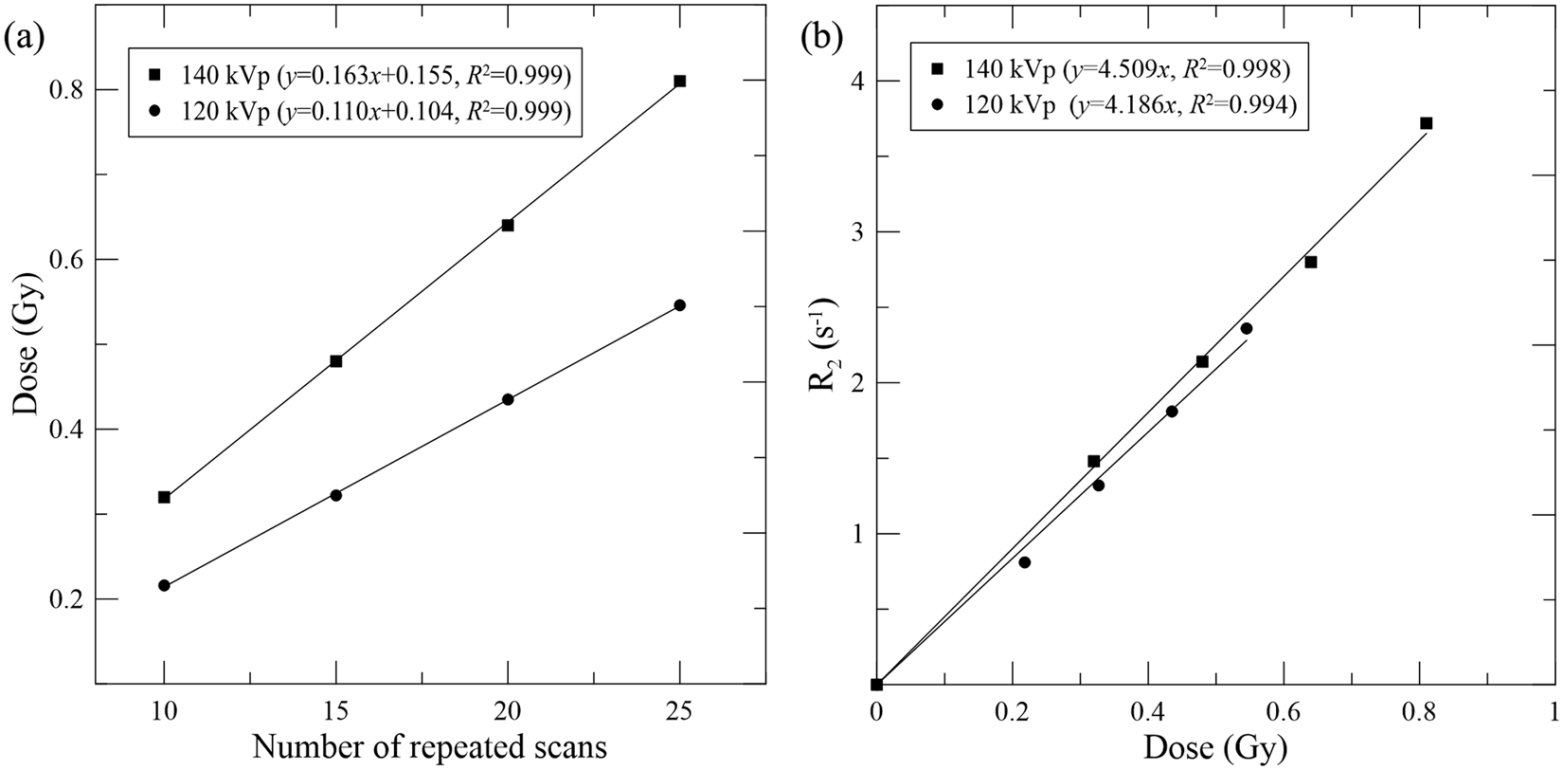
Relationships (**a**) between the dose and the number of repeated scans measured by the Farmer chamber, and (**b**) between the R_2_ value and the dose measured by the nMAG gel dosimeter under 120 and 140 kVp, respectively.

Figure 2(a) shows the measured dose for different numbers of repeated scans with the Farmer-type ionization chamber under 120 and 140 kVp, respectively. The dose measurements had a strong positive correlation with the number of repeated scans. The linearity of response curves was larger than 0.999. Figure 2(b) shows the fitting of R_2_ versus dose for the two tube voltages measured by the nMAG gel dosimeter. The dose sensitivities for 120 and 140 kVp were 4.186 Gy^−1^s^−1^, and 4.509 Gy^−1^s^−1^, correspondingly, indicating relatively small energy dependence of the nMAG gel in the energy range of CT scans. We further used these dose response curves for subsequent dose conversion.

### CTDI measurements

Tables 1 and 2 list the CTDI_100_ measured by the nMAG gel dosimeter and the 10-cm ionization chamber for different tube voltages and slice thicknesses. The tube current was fixed at 320 mAs. All CTDI measurements at the periphery were larger than those at the center. The main reason is that the center of the peripheral holes is only 1 cm away from the edge of the phantom, where the attenuation of photon beams is relatively low. The results obtained by the nMAG gel and the ionization chamber had a maximum difference of 5.29% for CTDI_100,c_, while the maximum difference was 1.82% for CTDI_100,p_. The differences for CTDI_100,w_ ranged from −1.21% to 0.83%, showing consistent results between both dosimeters.

**Table 1:** CTDI_100_ of different slice thicknesses measured by the 10-cm ionization chamber and the nMAG gel dosimeter at 120 kVp.

CTDI (mGy)	2 mm	5 mm	10 mm	14.4 mm	38.4 mm
CTDI_100,c_					
Ionization chamber	35.80	33.13	33.62	42.95	39.86
nMAG gel	33.92	31.53	32.20	44.47	41.97
Difference	−5.25%	−4.83%	−4.22%	3.54%	5.29%
CTDI_100,p_					
Ionization chamber	37.00	34.39	34.70	44.96	43.33
nMAG gel	37.47	34.79	35.33	44.58	42.81
Difference	1.27%	1.16%	1.82%	−0.85%	−1.20%
CTDI_100,w_					
Ionization chamber	36.60	33.97	34.34	44.29	42.18
nMAG gel	36.29	33.70	34.29	44.54	42.53
Difference	−0.85%	−0.79%	−0.15%	0.56%	0.83%

**Table 2:** CTDI_100_ of different slice thicknesses measured by the 10-cm ionization chamber and the nMAG gel dosimeter at 140 kVp.

CTDI (mGy)	2 mm	5 mm	10 mm	14.4 mm	38.4 mm
CTDI_100,c_					
Ionization chamber	50.42	46.30	46.86	60.01	55.00
nMAG gel	47.86	44.84	45.45	60.74	57.47
Difference	−5.08%	−3.15%	−3.01%	1.22%	4.49%
CTDI_100,p_					
Ionization chamber	51.57	47.57	47.54	62.53	58.73
nMAG gel	52.21	48.43	48.06	62.35	58.11
Difference	1.24%	1.81%	1.09%	−0.29%	−1.06%
CTDI_100,w_					
Ionization chamber	51.18	47.14	47.31	61.69	57.49
nMAG gel	50.76	46.57	47.19	61.81	57.90
Difference	−0.82%	−1.21%	−0.25%	0.19%	0.71%

Table 3 shows the CTDI_eff_ of different slice thicknesses measured for tube voltages of 120 kVp and 140 kVp. The results were all between 80.1%–82.5%, indicating an insignificant impact of the change in the tube voltage and slice thickness on the CTDI efficiency. The observed trend is consistent with the results obtained by Li *et al*.^7^.

**Table 3:** CTDI_eff_ of different slice thicknesses measured by the nMAG gel dosimeter at 120 kVp and 140 kVp.

Tube voltage	2 mm	5 mm	10 mm	14.4 mm	38.4 mm
120 kVp	80.4%	81.7%	81.2%	80.3%	82.5%
140 kVp	81.1%	81.6%	80.1%	80.4%	81.7%

### SSP measurements

**Figure 3:**
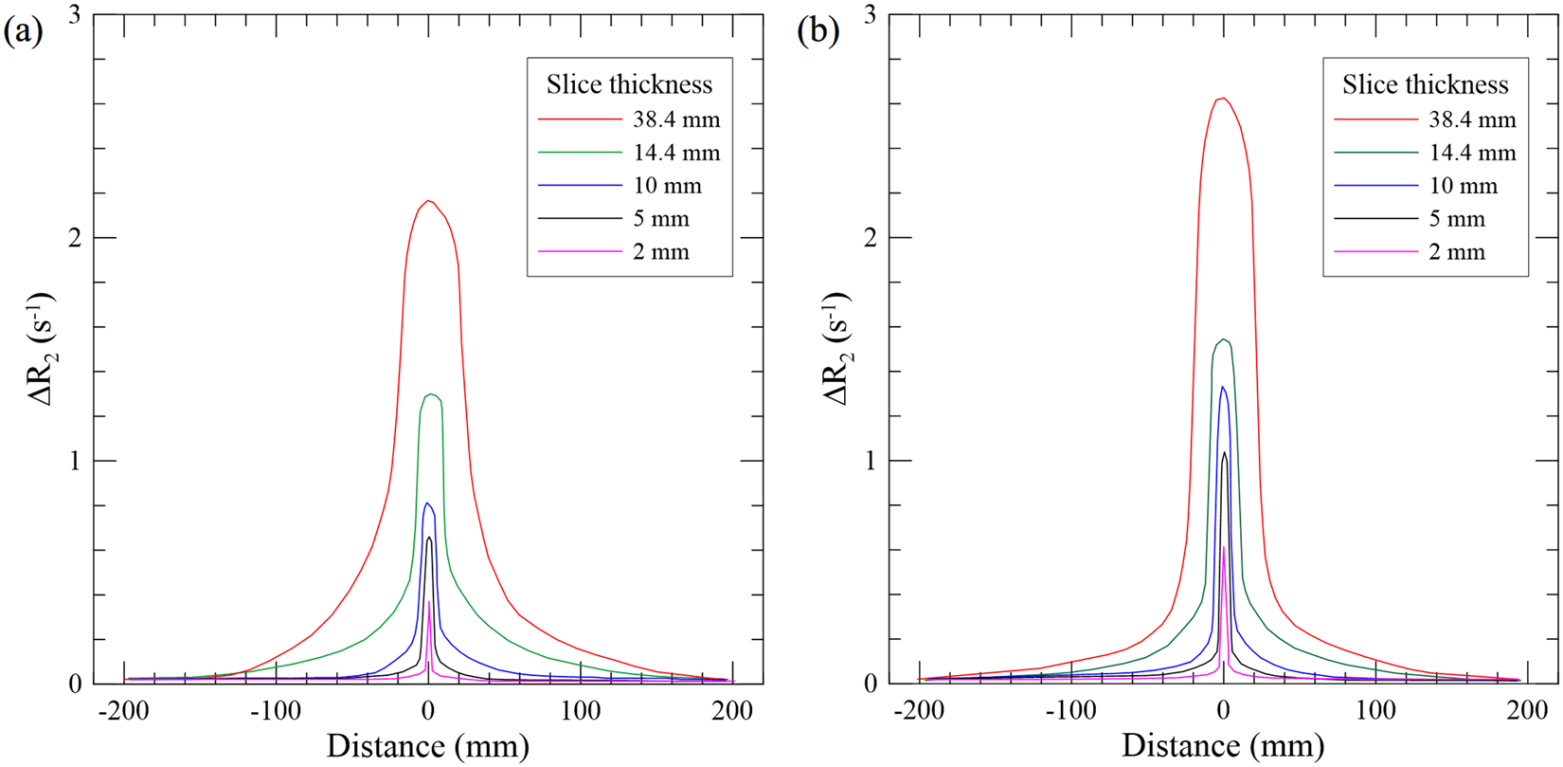
Dose profiles along the Z-axis under the slice thickness of 2, 5, 10, 14.4, and 38.4 mm at (**a**) center and (**b**) periphery of the PMMA phantoms.

Figure 3 shows the R_2_ profiles along the Z-axis for 120 kVp obtained by the nMAG gel dosimeter at the center and periphery (12 o’clock direction) locations. As the slice thickness increased, the scatters on both sides increased as well. In addition, the profile measured at the center showed larger scatter tails compared to that obtained at the periphery for the same slice thickness. The main reason for this is that the center position accumulates more in-scatters due to divergence of the beam, whereas the peripheral position accumulates more primary beams than scattered photons.

Table 4 shows the FWHM measured at the center and periphery locations using the nMAG gel dosimeter and Gafchromic film for various slice thicknesses. When the slice thickness increased, the difference between the measured FWHM and the nominal slice thickness increased. In addition, the FWHMs measured at the center were all larger than the corresponding FWHMs measured at the periphery. These results can be attributed to the contribution of scattered photons. When the Gafchromic films were used, the measured FWHMs were markedly greater than those measured by the nMAG gel dosimeters. This is especially pronounced in the center, where the difference measured by the film and gel significantly increased with the increase in the slice thickness. The FWHM measured by the film was up to 2.2 times the nominal slice thickness, whereas the results measured by the nMAG gel better represent the slice thickness.

**Table 4:** FWHM of different slice thicknesses measured by the nMAG gel dosimeter and Gafchromic film at 120 kVp.

FWHM (mm)	2 mm	5 mm	10 mm	14.4 mm	38.4 mm
Center					
Film	2.92	5.76	11.93	31.75	71.38
nMAG gel	2.54	5.46	10.66	19.81	48.02
Difference	13.0%	5.2%	10.6%	37.6%	32.7%
Periphery					
Film	2.57	5.28	10.06	18.39	47.48
nMAG gel	2.36	4.63	9.65	17.81	41.86
Difference	8.2%	12.3%	4.1%	3.2%	11.8%

## Discussion

In terms of CTDI_100,c_ measurements, the difference between the results obtained by the nMAG gel and the ionization chamber was less than 6%. Compared to the results obtained by Hill *et al*. who used polyacrylamide gel (PAG)^16,21^, the error was greatly reduced mainly because the nMAG gel has better dose sensitivities. Moreover, the fine slice thickness of 0.7 mm for MRI readouts was applied in order to obtain a higher spatial resolution for R_2_‒dose conversion. In terms of the MRI pulse sequence, 32 echo times were used, which produced more accurate T_2_ results than using eight echo times^22^. However, the disadvantage is a longer scanning time.

For CTDI_100,p_, the use of home-made PMMA phantoms provides the ability to simultaneously measure the CTDI at four peripheral locations with gel dosimeters. Even the weighted CTDI can be calculated after one CT exposure. For different slice thicknesses, the results measured by the nMAG gel and the ionization chamber matched very well. Since CTDI_100,p_ contributes two thirds of CTDI_100,w_, the error in weighted CTDI between the gel dosimeter and ionization chamber can be maintained within 1%. This verifies that the nMAG gel dosimeter can be used for output dose quality assurance in CT.

With the increase in CT slice thickness, the 150-mm-long commercial CTDI phantom is unable to cover enough scattered photons, leading to an underestimation of radiation dose in patients by using CTDI_100,w_. This study used three home-made PMMA phantoms and nMAG gel dosimeters to extend the measurement range to 450 mm, and further integrate the 300-mm dose profile to calculate CTDI_300,w_. The CTDI efficiency was in the range of 80.1%‒82.5%, and no significant dependence on kVp or slice thickness was observed. These results are consistent with the conclusion of Li *et al*.^7^ who used Monte Carlo simulations. Since CTDI_100,w_ is still used in clinical quality assurance and dose assessment, information given by the CTDI efficiency can be applied to correct CTDI_100,w_ to include the dose contributed from scattered radiation and to improve the accuracy of dose evaluation.

The characteristics of gel may affect the accuracy of dose and slice thickness evaluation. In terms of energy dependence, the effective atomic number is the most critical factor, especially in the energy range of diagnostic radiology. The nMAG gel has an effective atomic number of 7.6^23^, which is close to the effective atomic numbers of soft tissue (Z_eff_ = 7.64) and water (Z_eff_ = 7.51). The energy dependence of nMAG gel is less than 3% for photon energies between 6 MV and 25 MV^24^, which is comparable to a Farmer-type ionization chamber^25^. In this study, we observed mildly energy dependence of 7.2% for 120 kVp and 140 kVp in CT. Therefore, the degree of polymerization of nMAG gel caused by different tube voltages can be negligible. Among other radiation detectors, the effective atomic number of TLD is 8.31, which has a 14% variation in the range of 32–250 keV^26^. The EBT Gafchromic film has a Z_eff_ of 6.98, which also shows strong energy dependence in the range of 25 keV to 4 MV^27^. Therefore, the nMAG gel is more appropriate than other detectors for CT dose and image quality assurance.

The spatial resolution of a dosimeter is an important factor that affects dose profile and slice thickness assessment. Ionization chambers and TLDs, which are commonly used in clinical practice, are often limited by the size of their active volume and cannot produce continuous dose measurements. Although films and diode arrays can measure 2D dose distribution, they are difficult to incorporate with the CTDI phantom. The gel dosimeter provides 3D dose distribution, which makes it one of the most promising radiation detectors.

Other possible sources of error for this experiment are the preservation of gel and the MRI readouts. Studies have shown that exposure to high temperatures during gel preservation may cause colloid melting and diffusion problems^28^. Therefore, the irradiated gel tubes need to be directly placed in the MRI scanning room to avoid changes in temperature. In terms of MRI readouts, 1-hour scan time can raise the temperature of the gel tube by 2–4 °C due to the radio-frequency heating effect, which could also cause colloid instability. In addition, the gel tubes must be kept equidistant in all directions when placed in the water phantom for MRI readouts to avoid magnetic field inhomogeneity. The field uniformity should also be corrected for accurate T_2_ estimations^29^.

The major advantage in using the gel dosimeter and the home-made cylindrical phantoms for CT quality assurance is the extension of dose integration to 300 mm, making it possible to accurately assess the dose contribution from primary radiation and scattered photons. The weighted CTDI and the slice thickness can also be evaluated simultaneously, which has potential for clinical applications in MDCT and CBCT.

The main limitation of this experiment is the insufficient sensitivity of gel dosimeters. Although the nMAG gel is one of the most sensitive gel, the minimum dose of 100 mGy is still required to trigger polymerization. Therefore, CT scans must be repeated several times to accumulate sufficient radiation dose. In addition, the weighted CTDI cannot be assessed in conjunction with the 32-cm CTDI phantom or under 80-kVp tube voltage, since a significant portion of photons will be attenuated. These results should motivate the development of gel dosimeters with better dose sensitivities and the possible use of conversion factors to convert the dose measured in air (CTDI free-in-air) to the dose absorbed in the phantom (CTDI phantom)^30^.

## Conclusion

In this study, the nMAG gel dosimeter and the home-made cylindrical phantoms were used for CT dose and image quality assurance. In terms of radiation dose, the differences in CTDI_100,w_ obtained by the nMAG gel dosimeter and the 10-cm ionization chamber were less than 1%. The nMAG gel dosimeter can be used to measure CTDI_300,w_, and the results showed that the CTDI efficiency was in the range of 80.1%–82.5%, which can be used to correct the dose contributed from scattered photons in MDCT. In terms of image quality assurance, the nMAG gel dosimeter can be used to obtain the dose distribution along the Z-axis and calculate the FWHM of SSP for longitudinal resolution assessment. The weighted CTDI_300_ and SSP can be obtained at the same time, which shows potential for quality assurance and clinical applications in MDCT and CBCT.
